# Prediction of SARS-CoV Interaction with Host Proteins during Lung Aging Reveals a Potential Role for TRIB3 in COVID-19

**DOI:** 10.14336/AD.2020.1112

**Published:** 2021-02-01

**Authors:** Diogo de Moraes, Brunno Vivone Buquete Paiva, Sarah Santiloni Cury, Raissa Guimarães Ludwig, João Pessoa Araújo Junior, Marcelo Alves da Silva Mori, Robson Francisco Carvalho

**Affiliations:** ^1^Department of Structural and Functional Biology, Institute of Biosciences, São Paulo State University (UNESP), Botucatu, São Paulo, Brazil.; ^2^Faculty of Medicine, São Paulo State University, UNESP, Botucatu, São Paulo, Brazil.; ^3^Department of Chemical and Biological Sciences, Institute of Biosciences, São Paulo State University (UNESP), Botucatu, São Paulo, Brazil.; ^4^Department of Biochemistry and Tissue Biology, Institute of Biology, State University of Campinas (UNICAMP), Campinas, SP, Brazil.

**Keywords:** COVID-19, SARS-CoV-2, tribbles homolog 3, α-hydroxylinoleic acid, lung aging

## Abstract

COVID-19 is prevalent in the elderly. Old individuals are more likely to develop pneumonia and respiratory failure due to alveolar damage, suggesting that lung senescence may increase the susceptibility to SARS-CoV-2 infection and replication. Considering that human coronavirus (HCoVs; SARS-CoV-2 and SARS-CoV) require host cellular factors for infection and replication, we analyzed Genotype-Tissue Expression (GTEx) data to test whether lung aging is associated with transcriptional changes in human protein-coding genes that potentially interact with these viruses. We found decreased expression of the gene tribbles homolog 3 (*TRIB3*) during aging in male individuals, and its protein was predicted to interact with HCoVs nucleocapsid protein and RNA-dependent RNA polymerase. Using publicly available lung single-cell data, we found *TRIB3* expressed mainly in alveolar epithelial cells that express SARS-CoV-2 receptor ACE2. Functional enrichment analysis of age-related genes, in common with SARS-CoV-induced perturbations, revealed genes associated with the mitotic cell cycle and surfactant metabolism. Given that TRIB3 was previously reported to decrease virus infection and replication, the decreased expression of *TRIB3* in aged lungs may help explain why older male patients are related to more severe cases of the COVID-19. Thus, drugs that stimulate TRIB3 expression should be evaluated as a potential therapy for the disease.

The first cases of infections with the severe acute respiratory syndrome coronavirus 2 (SARS-CoV-2) in humans were identified in December 2019 in Wuhan, China [1, 2], and since then, the coronavirus disease 2019 (COVID-19) rapidly became pandemic [3]. Studies have shown that older individuals with comorbidities are associated with more severe cases of COVID-19 [4]. These patients are more likely to develop pneumonia and respiratory failure due to alveolar damage [5, 6], suggesting that lung aging impacts disease progression and mortality.

SARS-CoV-2 requires host cellular factors for successful infection and replication [7]. For example, angiotensin-converting enzyme 2 (ACE2) is the receptor for the SARS-CoV-2 spike protein receptor-binding domain (RBD) for viral attachment [7, 8]. The conserved evolutionary relationship between the 2019 novel SARS-CoV-2 and SARS-CoV [9] opens up the possibility to explore the relationships between these human coronaviruses (HCoVs) in public databases. Computational predictions of SARS-CoV-human protein-protein interactions (PPIs) may identify viral infection mechanisms and drug targets [9-11]. In this context, the Genotype-Tissue Expression (GTEx) database [12, 13] has provided insights into age-related genes [14, 15] and, associated with single-cell transcriptomics, could predict SARS-CoV-2-PPIs in aging lungs. This characterization is crucial for older adults, which are more vulnerable to the disease [16-18]. Thus, we analyzed whether lung aging is associated with transcriptional changes in proteins that potentially interact with SARS-CoV-2.

**Figure 1.**
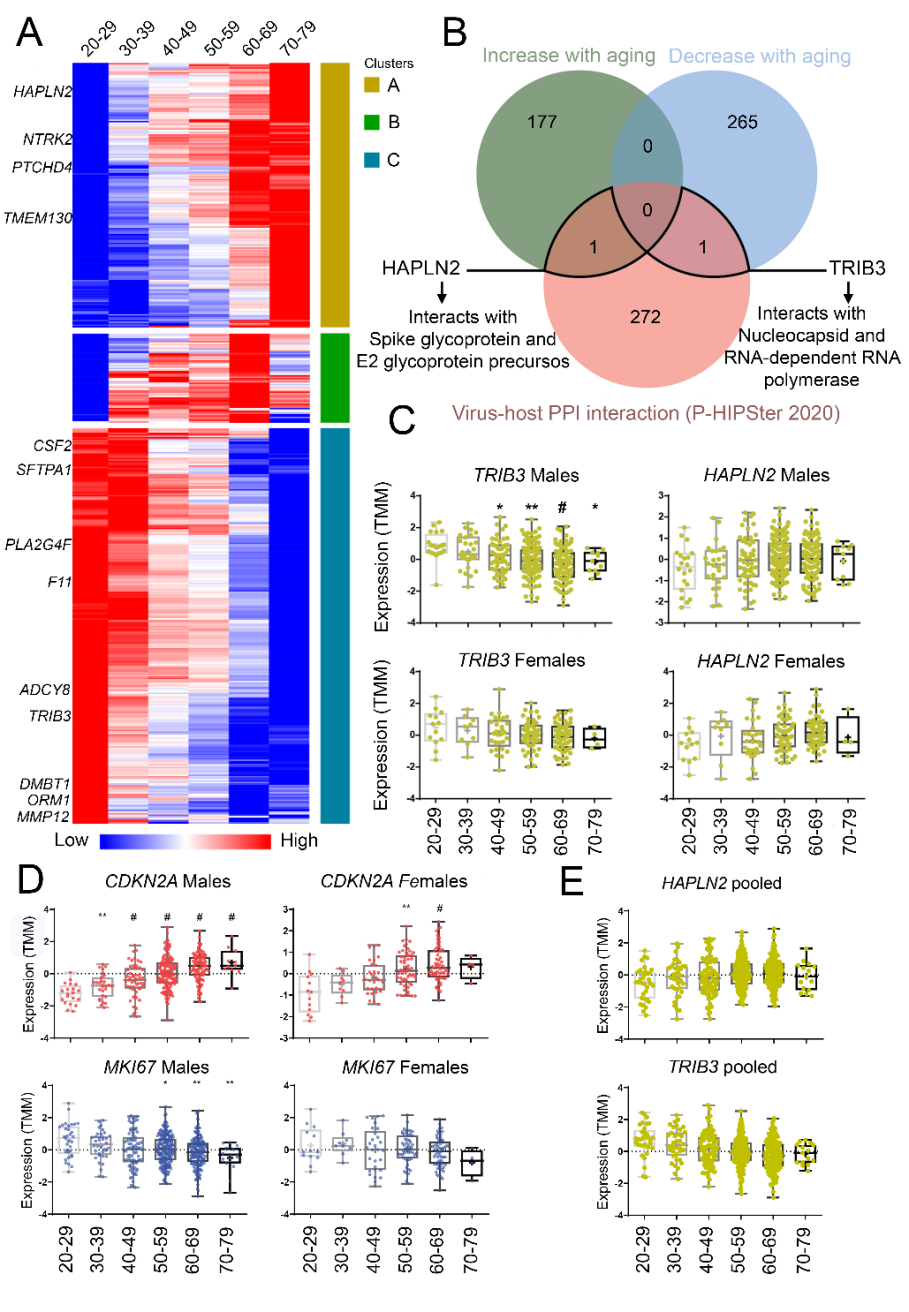
Lung gene expression of *TRIB3*, which is translated into a protein that potentially interacts with SARS-CoV-2 proteins, decreases in male individuals during aging. (A) Heatmap of mean TMM (Trimmed Mean of M-values) expression of males found as differentially expressed (DEGs; mean expression) in, at least, one age-group when compared to young adults (20-29 yo). These DEGs were found with males and females pooled. Rows were clustered using Euclidian distance. Clusters A and C contain genes that increase or decrease with age, respectively. *TRIB3*, *HAPLN2*, and the top 5 DEGs found in each age range are highlighted. (B) Venn diagram of DEGs during aging shared with the corresponding proteins that potentially interact with SARS-CoV-2 (arrows). Boxplots of gene expression levels (TMM) of separated or pooled sexes (C and E). Boxplot detailing the expression of genes that are well-recognized biomarkers of cellular division and senescence *CDKN2A* and *MKI67* in the lung (D). * P < 0.05, ** P < 0.001, and # P < 0.0001: statistical significance vs. young adults from GTEx v8 for Dunn’s test (males: 20-29 yo (n = 20), 30-39 yo (n = 27), 40-49 yo (n = 53), 50-59 yo (n = 135), 60-69 yo (n = 103), 70-79 yo (n = 11); females: 20-29 yo (n = 13), 30-39 yo (n = 9), 40-49 yo (n = 29), 50-59 yo (n = 52), 60-69 yo (n = 59), 70-79 yo (n = 4).

## METHODS

### Genes differentially expressed in GTEx lung samples during aging

The RNA-Seq data analysis of lung tissues was performed using 286 samples from males and 141 samples from females with 20-79 years old (yo) available at the GTEx portal (release V7) (https://www.gtexportal.org/)[19] (males: 20-29 yo (n = 16), 30-39 yo (n = 21), 40-49 yo (n = 47), 50-59 yo (n = 109), 60-69 yo (n = 86), 70-79 yo (n = 7); females: 20-29 yo (n = 11), 30-39 yo (n = 9), 40-49 yo (n = 29), 50-59 yo (n = 36), 60-69 yo (n = 53), 70-79 yo (n = 3)). We first used the BioJupies platform (https://amp.pharm.mssm.edu/biojupies/) [20] to identify the differentially expressed genes (DEG) in lung samples across age ranges using *limma* [21]. In this first analysis, only age, and not sex, was considered. The expression data of each subject was distributed according to their age range: 30-39; 40-49; 50-59; 60-69 and 70-79 yo, and each age range was compared with the group of young adults (20-29 yo) as a common control. Genes with Log2 of fold change ≥ |1| and false discovery rate (FDR) < 0.05 were considered as differentially expressed (DEGs) (Supplementary Fig. 1).

Next, for each sex, we performed a hierarchical clustering analysis based on Pearson correlation in age ranges using the mean expression (Trimmed Mean of M-values, TMM) of the DEGs found previously in, at least, one age range (Fig. 1A). This analysis aimed to identify clusters with gradients of gene expression across age ranges, showing increased or decreased expression during aging (method adapted from Theunissen et al., 2011 [22]). The clusterization of gene expression profiles on female lung samples generated clusters with less evident gradients than the males (Fig. 1A and Supplementary Fig. 2). Thus, considering the clear clusterization found in males and the fact that older males seem to have a worse prognosis on COVID-19 [6], we focused on male profiles to identify age-related lung genes.

Our research group previously used the strategy described above to analyze RNA-Seq data of GTEx lung samples (release V7) during aging. We reutilized these results due to the urgency of the current pandemic situation. GTEx was recently updated (V8), with more lung samples (males: 20-29 yo (n = 20), 30-39 yo (n = 27), 40-49 yo (n = 53), 50-59 yo (n = 135), 60-69 yo (n = 103), 70-79 yo (n = 11); females: 20-29 yo (n = 13), 30-39 yo (n = 9), 40-49 yo (n = 29), 50-59 yo (n = 52), 60-69 yo (n = 59), 70-79 yo (n = 4) (Supplementary Table 10). The updated cohort (V8) was used for further gene-specific analyses (*TRIB3*, *HAPLN2*, *CDKN2A*, and *MKI67*), with Dunn’s multiple comparisons test (Fig. 1C and E), through the GraphPad Prism version 8.0.0 for Windows (GraphPad Software, La Jolla, California, USA). P-values < 0.05 were considered as statistically significant.

### Predicted virus-host protein-protein interactions based on lung genes that increase or decrease expression during aging and SARS-CoV-induced perturbations

The conserved evolutionary relationship between the 2019 novel SARS-CoV-2 and SARS-CoV [9] opens up the possibility to explore relationships of these human coronaviruses (HCoVs) in publicly available databases. Thus, lung DEGs were compared with corresponding human proteins that potentially interact with HCoVs (Fig. 1B and Supplementary Table 2). The HCoVs-human PPIs were obtained using data from the Pathogen-Host Interactome Prediction using Structure Similarity (P-HIPSTer, http://phipster.org/) database, which is a catalog of the virus-human PPIs predicted based on protein structural information [11] (Supplementary Table 2) with an experimental validation rate of -76% [11]. The DEGs list were compared with recently added libraries for virus perturbations (up- and down-regulation) from GEO datasets (GSE33266, GSE50000, GSE49262, GSE50878, GSE49263, GSE40824, GSE50878, GSE49263, GSE47960, GSE47961, GSE47962, GSE17400, and GSE40824), available at the EnrichR database [23]. Access in March 2020. Genes that were up- or down-regulated in both conditions were analyzed on STRING (https://string-db.org/) [24]. Access in March 2020.

### Single-cell analysis of human lung datasets

Expression of *TRIB3*, *HAPLN2*, and *ACE2* was analyzed in different lung cell populations using two previously published human single-cell RNA-seq data (Supplementary Table 4) [25, 26]. The first dataset [25] was explored in the UCSC Cell Browser (http://nupulmonary.org/resources/), aiming the identification of the cell populations expressing those genes. The samples with pulmonary fibrosis presented in this dataset were omitted from our analysis, and only non-diseased lung samples were included (n=8). Another independent single-cell RNA-seq dataset [26] (n=5), available at the Human Cell Atlas Portal (https://data.humancellatlas.org/explore/projects/c4077b3c-5c98-4d26-a614-246d12c2e5d7), was used to confirm that *TRIB3* and *ACE2* are expressed in alveolar epithelial cells (types 1 and 2) and in ciliate cells.

### Protein-Protein Interactions (PPI) networks based on lung genes that increase or decrease expression during aging

The corresponding proteins of the DEG shared with the list of DEG from libraries for virus perturbations were queried in the STRING [24], for the construction of PPI networks. We considered the following settings: text mining, experiments, databases, and co-expression as sources of active interaction. We selected the minimum interaction score of 0.900 (highest confidence), and the disconnected nodes were hidden to simplify the display (Fig. 3B). We evaluated the PPI enrichment P-values, which verifies the number of interactions of a set of proteins compared with a random set of similar size. The PPI enrichment P-value represents the statistical significance provided by STRING. Access in March 2020.

### TRIB3 gene expression in lung samples from patients with comorbidities associated with severe COVID-19

To verify whether *TRIB3* expression is also decreased in the lungs of patients with comorbidities at high risk of developing severe COVID-19 such as asthma [27] and chronic obstructive pulmonary disease (COPD) [28], data of available studies were downloaded from public Gene Expression Omnibus (GEO) datasets (GSE158752, GSE85567 and GSE57148). Differential gene expression analysis was performed using the BioJupies platform by comparing each group of patients with asthma or COPD with their respective control group: 98 COPD vs. 91 controls, 57 Asthma vs. 28 controls, and 50 asthma vs. 17 controls. (Supplementary Fig. 3).

### Data representation and analysis

The clustering analyses of the expression profiles were performed using the web tool Morpheus (https://software.broadinstitute.org/morpheus)[29]. Venn diagrams were plotted using the Jvenn online tool (https://jvenn.toulouse.inra.fr)[30]. Volcano Plots were constructed with Graphpad Prism8.

**Figure 2.**
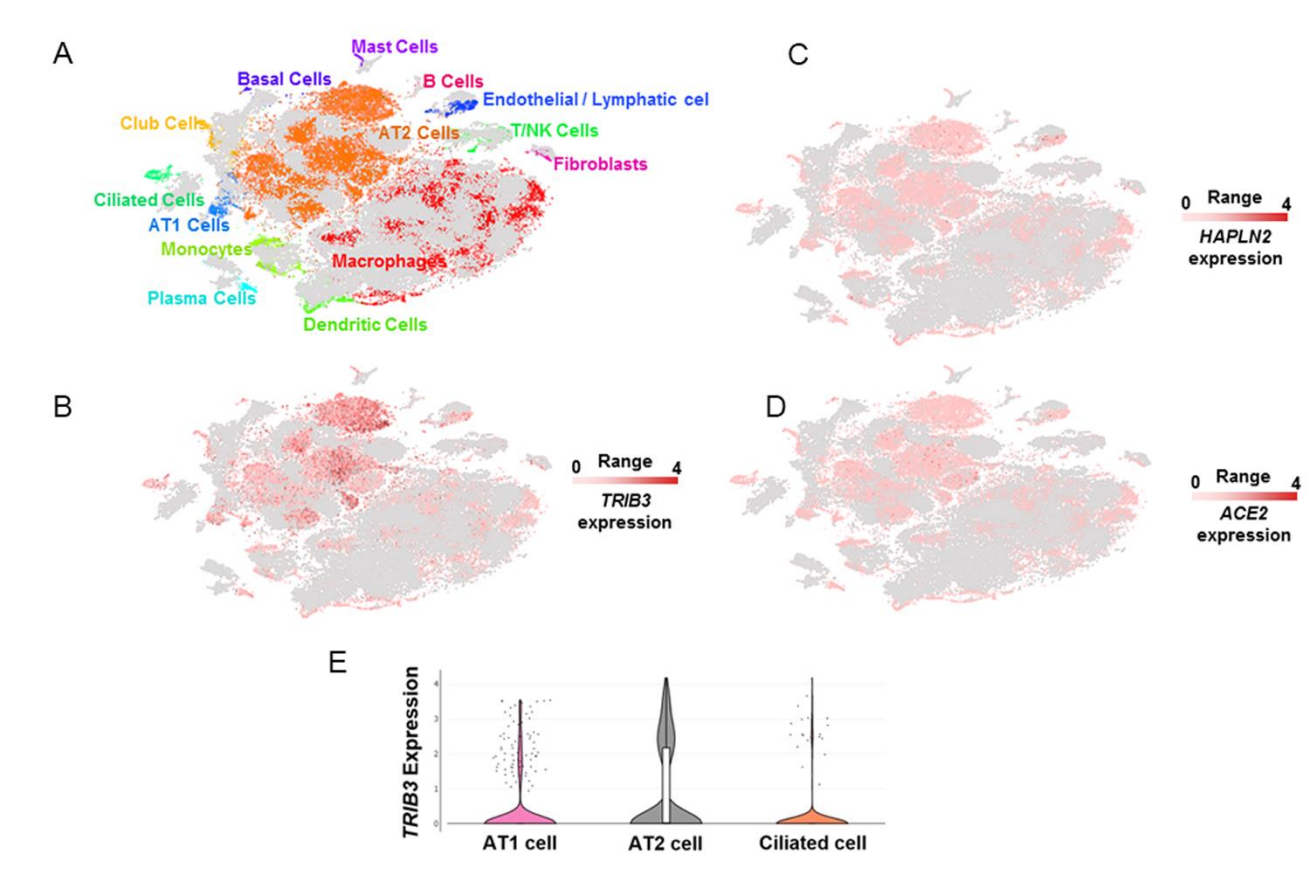
Single-cell gene expression analyses of *TRIB3*, *HAPLN2*, and *ACE2* in lung cells. (A) Unsupervised clustering demonstrates different cell populations identified in non-diseased lung human samples in a *t*-distributed Stochastic Neighbor Embedding (tSNE) plot, as described previously [25]. Grey dots represent single cells from pulmonary fibrosis samples that were not included in the present analysis. Single-cell gene expression of *TRIB3* (B), *HAPLN2* (C), and *ACE2* (D) in different lung cell populations. The images were generated using the dataset [25], available at nupulmonary.org/resources/. The range represents the minimum and maximum expression. (E) Violin plots of *TRIB3* expression levels in lung single-cells.

## RESULTS

We identified differentially expressed genes (DEGs) during aging in GTEx human lung samples (release V7) (Data S1). The numbers of significant DEGs increased with aging (Log fold-change ≥ |1| and FDR < 0.05), and individuals of 60-69-year-old (yo) presented the highest number of DEGs, in comparison to young adults (20-20 yo) (Figures S1-S2, Table S1). Clustering of these DEGs identified age-associated profiles (Figure 1A). Among the transcripts translated into proteins predicted as interacting with SARS-CoV, the hyaluronan and proteoglycan link protein 2 (*HAPLN2*) increased with aging, while tribbles homolog 3 (*TRIB3*) decreased (Fig. 1B, Supplementary Table 2). HAPLN2 was predicted to interact with virus proteins spike glycoprotein and E2 glycoprotein precursors, while TRIB3 with nucleocapsid protein and RNA-dependent RNA polymerase (Fig. 1B; Supplementary Table 3). Notably, the SARS-CoV-2 nucleocapsid protein has a sequence identity of 89.6% compared to SARS-CoV [9]. The expression of *TRIB3* also decreased in the lung, specifically in males older than 40 (Fig. 1C), in a cohort with additional samples (GTEx, release V8). When both sexes are pooled, *HAPLN2* expression is significantly increased in individuals older than 60 (Fig. 1E).

**Figure 3.**
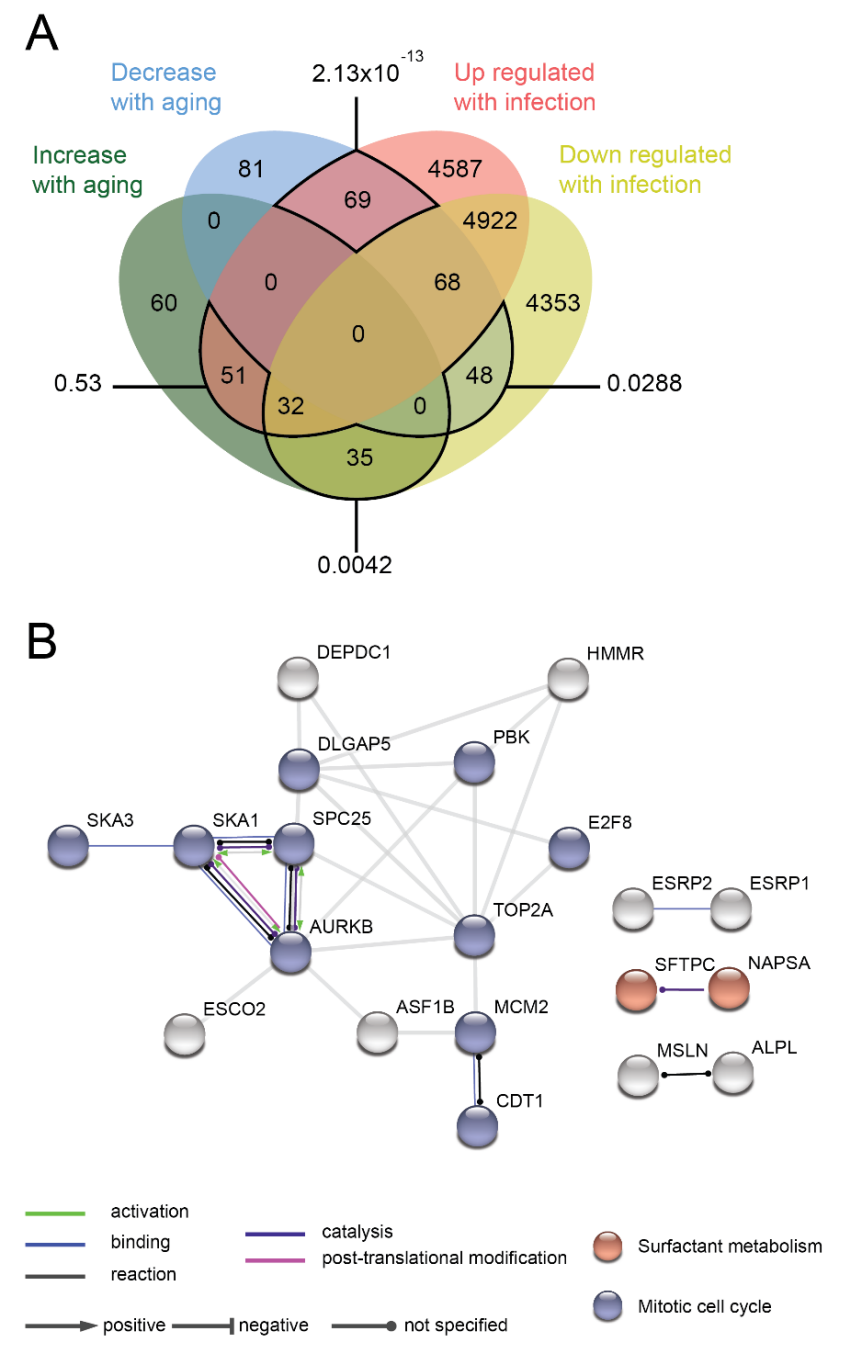
Over-represented genes altered in aging and SARS-CoV infection are associated with mitotic cell cycle and surfactant metabolism. (A) Venn diagram of differentially expressed genes (corresponding proteins) during aging shared with SARS-CoV-induced perturbations in host gene expression. Values outside the diagram: PPI enrichment P-value. (B) PPI network based on the genes that decreased expression during aging and are up-regulated in SARS-CoV-induced perturbations in host gene expression. Table S8 contains the complete list of over-represented terms.

We also measured the lung expression profile of the well-known aging markers *CDKN2A* and *MKI67* in GTEx human lung samples [14, 31-33]. *CDKN2A* is translated into the INK4 family members p16 and p16(INK4a), which are markers of senescence and physiological aging [1-3]. *MKI67* is a cellular marker of proliferation, and its absence indicates senescence [4]. *CDKN2A* significantly increased with aging in both sexes, whereas *MKI67* decreased in males (Fig. 1D).

The reanalysis of lung single-cell RNA sequencing data [25, 26] demonstrated that *TRIB3* was expressed mainly in alveolar type I (AT1) and type II (AT2) cells and in ciliated cells (Fig. 2A, D and E), which also expresses the SARS-CoV-2 receptor ACE2 [7, 8, 34].

*TRIB3* expression was decreased in lung comorbidities associated with COVID-19 severity. In COPD patients, *TRIB3* showed logFC = -0.32 and p<0.001 and, for asthma patients, *TRIB3* were reduced in both datasets (logFC = -0.3 and -0.4, p<0.05). We compared the young asthma patients (<35 years-old; N=20) with the old asthma patients (>50 years-old; N=21) from GSE158752. Even showing negative logFC expression (-0.28) the difference was not significant (Suppmentary Fig. 3). Finally, we compared SARS-CoV-induced perturbations in host gene expression, from public GEO datasets, with our list of DEGs in GTEx lung samples during aging (Fig. 3, Supplementary Table 8). We found that genes that decrease their expression with aging and genes that are up-regulated with SARS-CoV infections generated the most significant network, with over-represented genes associated with mitotic cell cycle and surfactant metabolism (Fig. 3B).

## DISCUSSION

Here, we used the transcriptome of lung samples from the GTEx database to find age-related genes. To predict how these genes could interact with SARS-CoV-2 infection, we used two approaches: predicted their interaction with the proteins of the closely related SARS-CoVs strain through the P-Hipster database(i); checked if these genes were also deregulated on SARS-CoV-2 infections through the EnrichR database (ii).

The involvement of TRIB3 in viral infection is poorly understood; however, its inhibition was associated with an increase of hepatitis C virus (HCV) replication [35]. Additionally, TRIB3 negatively regulates the entry step of the HCV life cycle and propagation [35] and may constitute a common protective host factor for other positive-sense single-strand RNA viruses. *TRIB3* is also one of the unfolded protein response (UPR)-related genes with the strongest positive correlation with the intracellular abundance of the flavivirus dengue and Zika [36]. Considering the need for drugs to treat COVID-19, the α-hydroxylinoleic acid (ABTL0812) induces the expression of TRIB3 by inhibiting the PI3K/AKT/mTOR axis and promoting autophagy cell death in cancer [37]. We highlight that the lifecycle of coronaviruses depends on several host-cell encoded cellular pathways, and among these pathways, UPR and autophagy pathways of the host cells are essential to the life cycle of coronaviruses [38].

We also found opposite functional directions of mitosis and surfactant metabolism in aging lungs when compared to SARS-CoV-2-induced perturbations. The decreased cellular division capacity on aging is associated with cellular senescence - a mechanism that stops cells with damaged DNA from replicating [39] - and progenitor cell exhaustion [40]. The altered metabolism or secretion of surfactants by AT2 cells reduces the ability of the lungs to expand and increases the risk of alveolar collapse in HCoVs infections [41, 42]. Moreover, *Sftpc*^-/-^ (Surfactant Protein C) mice have worse viral infections than controls [43], and its human homolog decreased with aging while it is up-regulated on SARS-CoV infections (Fig. 3). Thus, the pneumonia-like lung injury found in severe cases of COVID-19 infections [5, 6] may be aggravated by impaired lung regeneration and altered metabolism of surfactants in older male patients.

Although the genes and pathways we highlighted were identified based on robust statistical significance, other methods of over-time gene expression analyses applying different cutoffs could be considered; using GTEx V8 cohort or separating males and females may result in different sets of age-related genes in the lung. Further analyses should be conducted to identify more differences between male and female lungs during aging. Additionally, clinical data from these individuals - such as diabetes or cardiovascular diseases - important factors influencing COVID-19 outcome - were not evaluated. However, the GTEx donor consent policy makes public phenotypes limited. Its access needs an application via dbGaP (Genotypes and Phenotypes database), which, associated with reanalysis of the transcriptomics data, may take significant time. Part of the results presented herein derives from a previously unpublished paper focusing on aging lung on a different topic. Nevertheless, we decided to release this data focusing on SARS-CoV-2 due to the emergency of the current pandemic.

In conclusion, we show that lung gene expression of TRIB3, a protein predicted to interact with the nucleocapsid protein and the RNA-dependent RNA polymerase of HCoVs, decreases in COPD, asthma and males during aging. This study provides insights into aging and COVID-19 based on the transcriptional profile of the aging lung and reveals a potential role for TRIB3, surfactant metabolism, and mitotic cell cycle. Considering that TRIB3 may decrease virus infection and replication, strategies to stimulate TRIB3 expression should be tested to treat COVID-19.

## Supplementary Materials

The Supplementary data can be found online at: www.aginganddisease.org/EN/10.14336/AD.2020.1112. Other supplementary files can be found in the following repository: https://zenodo.org/record/4122440. DOI: 10.5281/zenodo.4122440.


