# Supplementary material for: Prediction of SARS-CoV Interaction with Host Proteins during Lung Aging Reveals a Potential Role for TRIB3 in COVID-19
Source: Aging Dis. 2021 Feb 1;12(1):42–9. doi: 10.14336/AD.2020.1112 (PMC7801268; doi:10.14336/AD.2020.1112)
Supplement: Supplementary file 1 [file AD-12-1-42-S.pdf]

SUPPLEMENTARY DATA

Prediction of SARS-CoV Interaction with Host Proteins during Lung Aging Reveals a Potential Role for TRIB3 in COVID-19

Diogo de Moraes[1#], Brunno Vivone Buquete Paiva[1,2,#], Sarah Santiloni Cury[1], Raissa Guimarães Ludwig[4], João Pessoa Araújo Junior[3], Marcelo Alves da Silva Mori[4], Robson Francisco Carvalho[1*]

© 2020. Moraes DD et al. Published online at http://www.aginganddisease.org/EN/10.14336/AD.2020.1112

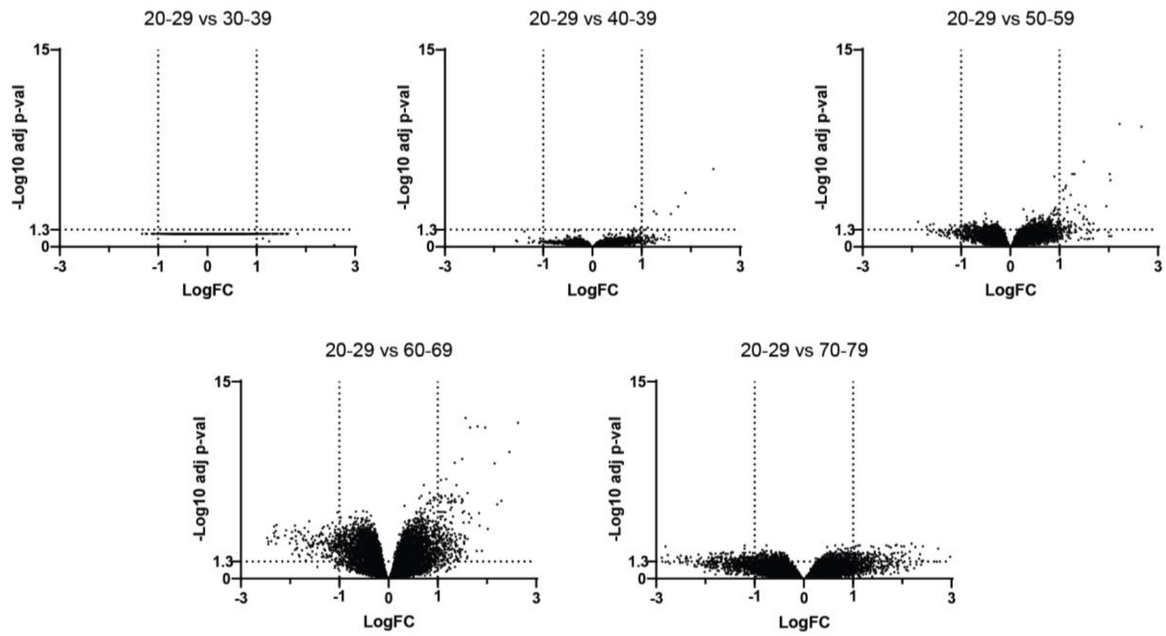

Supplementary Figure 1. Volcano plot showing lung transcriptional deregulation in 427 GTEx male and female samples during aging, represented as −log10 (adjusted p-value) and log fold change difference. Dashed lines represent our cutoffs (LogFC ≥ |1| and FDR<0.05). The differentially expressed genes (DEG) with age were identified by distributing the expression data of each individual into five age groups (30-39; 40-49; 50-59; 60-69 and 70-79 years old) and compared with a group of young individuals (20-29 years old). Number of men: 20-29 yo (n = 16), 30-39 yo (n = 21), 40-49 yo (n = 47), 50-59 yo (n = 109), 60-69 yo (n = 86), 70-79 yo (n = 7). Number of women: 20-29 yo (n = 11), 30-39 yo (n = 9), 40-49 yo (n = 29), 50-59 yo (n = 36), 60-69 yo (n = 53), 70-79 yo (n = 3). 7 genes were up regulated on 40-49 yo, 56 on 50-59 yo, 217 on 60-69 yo, and 144 on 70-79 yo. Eleven genes were down regulated on 50-59 yo, 179 on 60-69 yo, and 291 on 70-79.

© 2020. Moraes DD et al. Published online at http://www.aginganddisease.org/EN/10.14336/AD.2020.1112

SUPPLEMENTARY DATA

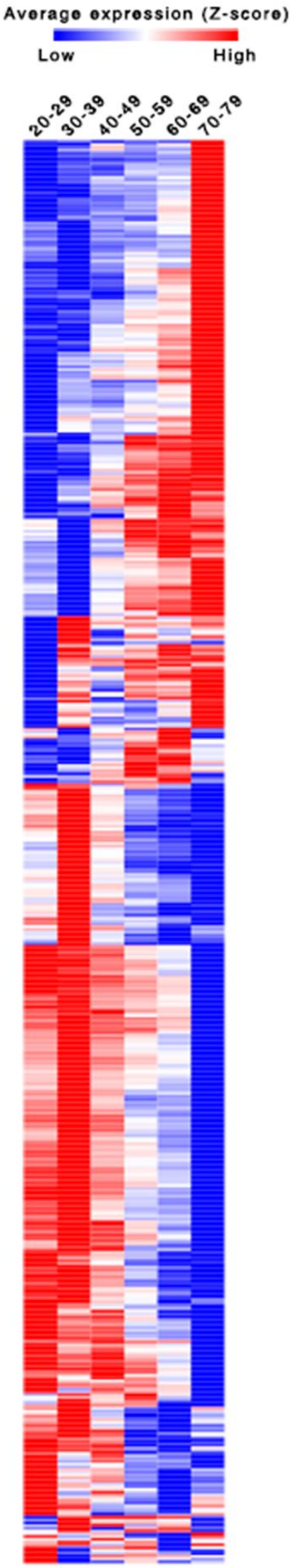

Supplementary Figure 2. Heatmap with the mean expression of DEGs on female samples (n=129), normalized by the Trimmed Mean of M-values (TMM) and Z-scored by row. Fewer genes are clustered on gradients than in males (Figure 1A), which could mean more noise on the data due to decreased sample size or female hormonal cycle.

© 2020. Moraes DD et al. Published online at http://www.aginganddisease.org/EN/10.14336/AD.2020.1112

SUPPLEMENTARY DATA

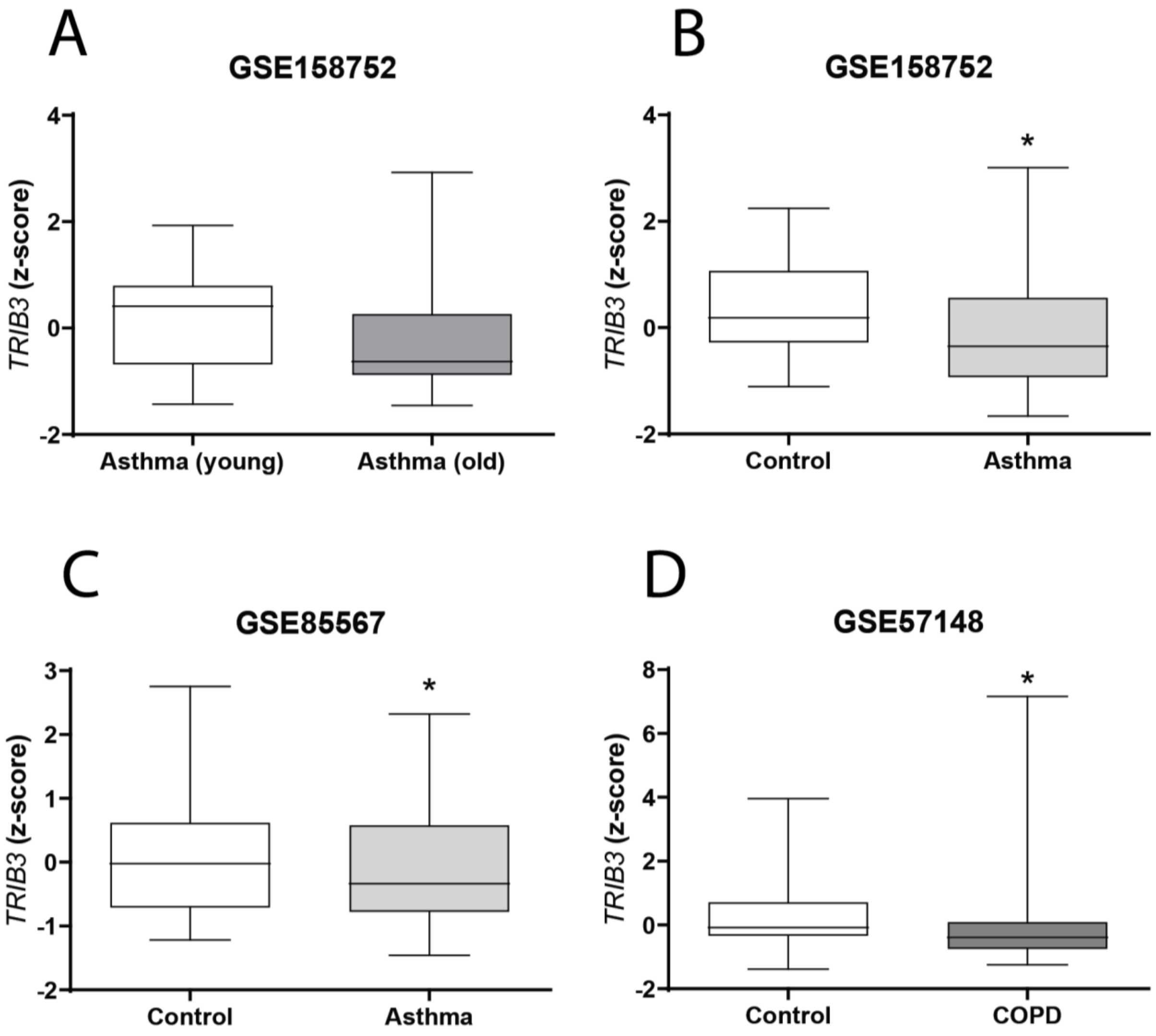

Supplementary Figure 3. *TRIB3* expression (RNA-seq) in lung from patients with comorbidities associated with severe COVID-19 retrieved from Gene Expression Omnibus (GEO). *TRIB3* expression is decreased in the lung of asthma patients from two independent cohorts (B and C), but not in young vs. old asthma patients (A). Patients with chronic obstructive pulmonary disease also have decreased expression of *TRIB3* (D). Differential gene expression analysis performed using Biojupies default settings. * indicates significant comparisons (p-value < 0.05).

© 2020. Moraes DD et al. Published online at http://www.aginganddisease.org/EN/10.14336/AD.2020.1112
